# Seasonal Variation in Type 1 Diabetes Incidence in Poland: Exploring the Impact of Viral Infections, Including COVID-19

**DOI:** 10.1155/pedi/6868987

**Published:** 2025-09-04

**Authors:** Daniel Matuszelański, Artur Winiarczuk, Mateusz Tuszyński, Marta Wysocka-Mincewicz, Zuzanna Piechnik, Lidia Groele, Agnieszka Szypowska

**Affiliations:** ^1^Department of Paediatric Diabetology and Paediatrics, Medical University of Warsaw, Warsaw, Poland; ^2^Clinic of Endocrinology and Diabetology, Children's Memorial Health Institute, Warsaw, Poland; ^3^Department of Inborn Errors of Metabolism and Paediatrics, Institute of Mother and Child, Warsaw, Poland

## Abstract

**Objective:** Seasonal variation in type 1 diabetes (T1D) incidence has long been a focus of epidemiological research, with viral infections among the proposed contributing factors. Our aim was to examine the seasonal pattern of T1D onset in Poland and to assess how viral infections—including COVID-19—may influence this seasonality.

**Methods:** We analyzed data from 2381 children with newly diagnosed T1D admitted to two pediatric diabetes centers in the Masovian Voivodeship between 2015 and 2023 and compared them with epidemiological data on COVID-19 and influenza cases during the same period.

**Results:** Our analysis revealed a 30% increase in T1D cases over the study period, with a pronounced seasonal pattern: the highest number of diagnoses occurred in February and the lowest was noted in June. Children under 4 years of age exhibited a distinct pattern with a peak in October, suggesting age-specific differences in T1D pathogenesis. Overall, T1D onset was more frequent in autumn–winter than in spring–summer, with 1294 (54%) vs. 1087 (46%) cases, respectively (*p*  < 0.0001). The influence of COVID-19 on T1D incidence was limited to the first wave of the pandemic. During this period, a strong association was observed (*r* = 0.96, *p*  < 0.001), whereas no correlation was found during the second wave (*r* = 0.086, *p* = 0.87). The seasonality of T1D diagnoses closely correlated with that of influenza infections (*r* = 0.79, *p* = 0.002). However, the overall trends differed, suggesting that other viruses with similar transmission patterns may contribute to the seasonality of T1D onset.

**Conclusion:** These findings underline the complex interplay between viral infections and T1D seasonality and suggest that public health strategies aimed at mitigating severe viral infections, including vaccination, warrant further investigation for their potential role in modulating T1D onset in susceptible individuals.

## 1. Introduction

Seasonal variation in the incidence of type 1 diabetes (T1D) mellitus has long been a subject of interest in epidemiological research. Seasonality in the clinical diagnosis of T1D has been observed in many countries [1–10], with the highest incidence reported in colder months and the lowest in warmer months. The magnitude of month-to-month differences varies depending on the study and geographic latitude; however, most studies are consistent with this finding. In our previous study, conducted between 2010 and 2014, we demonstrated the seasonality of T1D incidence in children in Poland. The majority of children were diagnosed in autumn and winter [6].

Numerous studies suggest that environmental factors, including seasonal changes, may influence the autoimmune processes leading to beta-cell destruction in genetically predisposed individuals. Hypotheses have linked viral infections [1, 3–7, 9, 11], sunlight exposure [3, 8], genetic factors [12], vitamin D levels [2, 3, 9], and intrauterine exposure to infections [13]. Among these, viral infections are the most frequently mentioned. Within this category, the association between enteroviruses and the development of T1D is the most extensively documented [14–16]. However, they exhibit a different seasonality than T1D, so their role in the observed seasonality of T1D incidence is uncertain. Other viral infections, particularly respiratory viruses, are more prevalent in colder months and may also contribute to T1D by triggering or accelerating autoimmunity [17].

Vitamin D deficiency also has been associated with the development of T1D. In Poland, vitamin D deficiency is a common issue [18, 19], especially in winter and spring. The main natural source of vitamin D is skin synthesis triggered by UVB radiation, but from October to April, UVB levels in Poland are too low to ensure sufficient production. However, the impact of vitamin D deficiency on the development of T1D remains uncertain [20].

The SARS-CoV-2 virus is a novel pathogen that may have had a significant impact on the seasonality of T1D incidence. The first SARS-CoV-2 case in Poland was reported in March 2020. Despite lockdowns, the virus spread rapidly, peaking in deaths between March and April 2021, with around 91,000 fatalities that year [21]. The data from the SWEET register, which included data from Poland, indicated a potential impact of the COVID-19 pandemic on the seasonality of T1D. However, that study analyzed data only through 2021 [22].

The aim of the study is to assess the seasonality of T1D incidence in children in the Masovian Voivodeship between 2015 and 2023. Furthermore, we aimed to explore the possible causes of the monthly fluctuations observed, focusing on the potential impact of viral infections.

## 2. Materials

From January 1, 2015, to December 31, 2023, 2400 children below 18 years of age with newly diagnosed T1D were admitted to two pediatric diabetology centers in the Masovian Voivodeship in Poland: the Medical University of Warsaw and the Children's Memorial Health Institute. Of the total cases, 1642 (68.42%) were from the Medical University of Warsaw Hospital and 758 (31.58%) from the Children's Memorial Hospital. Nineteen cases (0.79%) were excluded from the analysis due to incomplete data—missing exact admission date, sex, or birth date—resulting in a final sample of 2381 cases. Data were obtained from the hospital electronic database. The demographic characteristics of the study group showed a mean age of 9.38 years (±4.33 years); the majority were boys (1303 cases, 54.68%).

To analyze T1D incidence trends while accounting for demographics, we obtained data from the Central Statistical Office (GUS) [23] on the number of 10-year-olds. We selected the population of 10-year-old children as a reference group because 10 years represents the median age within our cohort, with the mean age (9.38 years) being close to this value, and also approximately marks the midpoint of the 0–18 age range. Considering these aspects, as well as the fact that demographic shifts are long-term processes, we assumed that this demographic index would help prevent misinterpretation of trends, such as attributing a decline in cases to actual disease reduction when it could result from a shrinking child population. The population for each year in the Masovian Voivodeship was estimated using past birth records, assuming minimal migration impact in this age group.

To accurately investigate potential causes of monthly variations in T1D diagnoses, the analysis focused on seasonal viral infections, recognized as a key factor. Due to the lack of a comprehensive database on all viral infections in Poland, the influenza incidence registry maintained by the National Institute of Public Health [24] was selected, as it best met the criteria of reflecting the local situation in the Masovian Voivodeship and being reported monthly. To ensure the analysis covered full years, data up to 2022 were included, since influenza data were available monthly only until June 2023.

Additionally, monthly average temperatures in Warsaw, provided by the Institute of Meteorology and Water Management (IMGW) [25], were analyzed to account for environmental factors, such as physical activity levels and sunlight exposure. Given that the study period overlapped with the COVID-19 pandemic, historical COVID-19 case data for the Masovian Voivodeship were also incorporated [21].

## 3. Methods

To assess the overall trend in new T1D diagnoses, annual case numbers were aggregated, and a linear regression model was applied. As outlined in the Materials section, to prevent misinterpretation of trends, demographic data were incorporated by calculating an index as follows:

  $$\begin{array}{c} \left(\frac{\text{Number of cases}\in \text{a given year}}{\text{Number of individuals at the median age of onset}\in \text{the Masovian Voivodeship}\in \text{the same year}}\right)\times 1000. \end{array}$$

This index was also analyzed using linear regression.

To examine seasonality, time series decomposition using the Census I method was applied, which separates data into trend-cycle, seasonal, and irregular components. Given the nature of the dataset, an additive model was used, with seasonal factors computed for each month. To compare T1D seasonality with influenza cases and average monthly temperatures in the Masovian Voivodeship, the same decomposition approach was applied to these datasets. Since lower temperatures have been previously linked to higher T1D incidence, negative temperature values were analyzed, assuming an inverse relationship between temperature and T1D diagnoses. The seasonal components were then visually compared, and correlation analysis was performed between the seasonal factors of the different datasets.

During the correlation analysis, Pearson's or Spearman's tests were used, depending on the fulfillment of the assumptions required to perform these tests on the given dataset.

Correlation analysis was also conducted between the number of monthly diagnoses of T1D and the number of COVID-19 cases during two identified waves of the COVID-19 pandemic. Prior to performing the correlation analysis, the datasets were plotted to identify potential outliers that could distort the results. This was the case only during the first wave of the COVID-19 pandemic. February 2021 was identified as an outlier. This is most likely due to the fact that February had the highest number of diabetes diagnoses throughout the entire study period, compounded by the concurrent impact of the COVID-19 pandemic, as discussed later. Consequently, February 2021 was excluded from the correlation analysis.

To identify months with significantly different diagnosis frequencies from expected values, the chi-square goodness-of-fit test was applied.

Previous studies suggest distinct monthly diagnosis patterns in the youngest age group (0–4 years) [5, 6, 9]. However, in our dataset, children aged 0–4 years accounted for only 19.19% (457 cases), with some months having zero diagnoses. Given the small sample size and the potential influence of random variation, reliable time series decomposition was not feasible. Therefore, for age-group comparisons, the analysis was limited to assessing the percentage contribution of each month to the total diagnoses.

All statistical analyses were conducted using Statistica version 14.00.15 (TIBCO Software Inc., 2020).

## 4. Results

**Figure 1:**
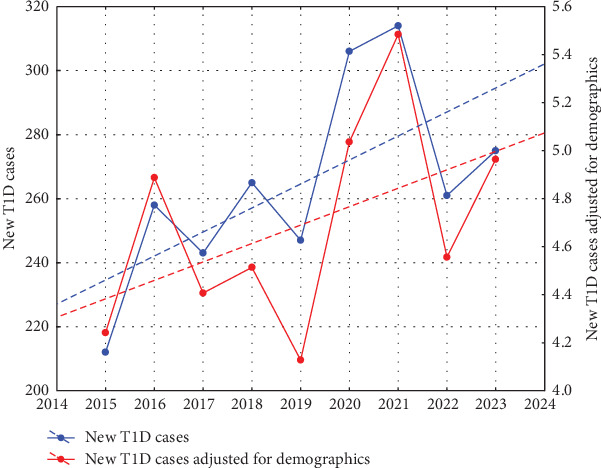
Annual incidence of new T1D cases. The blue dots represent the observed number of new diagnoses. Between 2015 and 2023, the number of new cases increased by approximately 30%. The highest incidence was recorded in 2021, with 314 cases. The linear model (blue dashed line) demonstrates a moderate fit to the data, without statistical significance. The red dots represent the number of new cases indexed to the 10-year-old population in the Masovian Voivodeship (cases per 1000 children aged 10). The linear model (red dashed line) shows an even weaker fit to the data, with a less pronounced upward trend.

We recorded an approximately 30% increase in children with newly diagnosed T1D in the Masovian Voivodeship between 2015 and 2023 (212 and 275 cases, respectively), with a peak noted in 2020 and 2021. The linear regression analysis of T1D diagnoses for the years 2015–2023 revealed an upward trend, with the model fit approaching, but not reaching, statistical significance (*R*^2^ = 0.4179, *p* = 0.0599). (Figure 1) The linear regression analysis of new cases adjusted for demographic factors showed a very slight upward trend. The linear model fit to the data was weaker (*R*^2^ = 0.2307, *p* = 0.1906).

**Figure 2:**
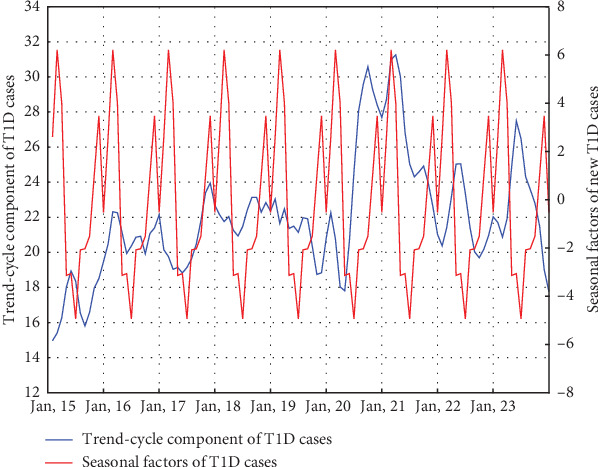
Time series decomposition of new T1D cases. Seasonal factors peak in February (6.206) and reach their lowest value in June (−4.94). Notably, seasonal factors do not increase steadily between June and February, as a pronounced slowdown is observed in December. The trend-cycle component remains relatively stable over time, with a noticeable increase between March 2020 and July 2021, peaking in March (31.26) and February (30.98) 2021.

The results of the time series decomposition for new T1D diagnoses, using the Census I method, revealed several important findings. The trend-cycle component remained relatively stable over time, with a notable peak between March 2020 and July 2021, reaching its highest point in March and February 2021. The seasonal component showed the highest seasonal factor of 6.206 in February and the lowest in June at −4.94. This indicates that, after removing long-term trends and random components from the data, the highest number of diagnoses occurred in February, while the lowest occurred in June. Additionally, the seasonal factor did not increase steadily from June to February, as there was a decrease in December, followed by a rise in January. (Figure 2)

**Figure 3:**
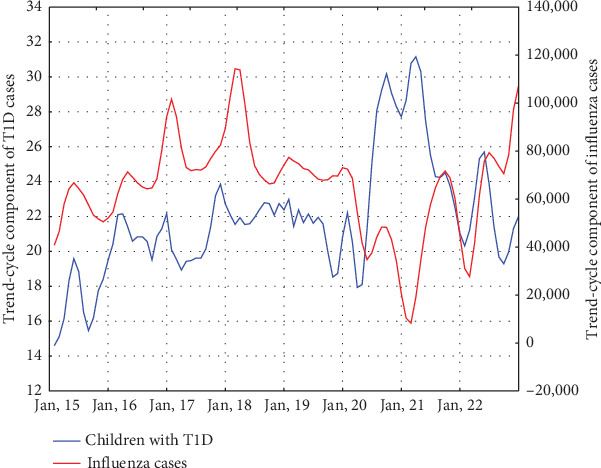
New cases of T1D and influenza—trend-cycle components. The trend-cycle components of both datasets remained relatively stable until 2020, when the number of new T1D cases began to rise, while influenza cases started to decline. In March and February 2021, a peak in T1D incidence was observed, coinciding with the lowest recorded number of influenza cases noted in February 2021 (trend-cycle components 30.77 and 8325.7, respectively).

**Figure 4:**
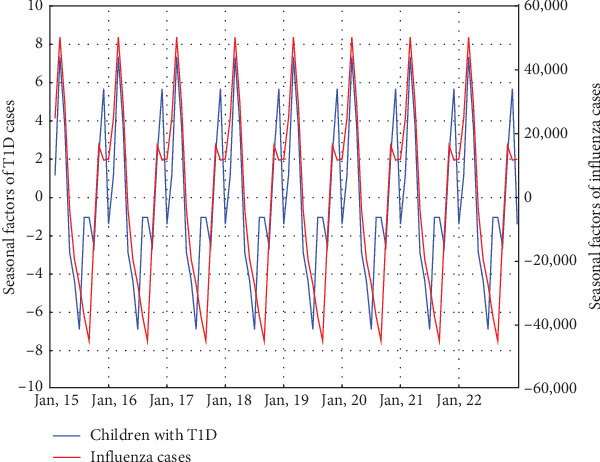
New cases of T1D and influenza—seasonal factors. Both T1D and influenza exhibit a similar seasonal pattern, with seasonal factors peaking in February and reaching their lowest values in summer—June for T1D and August for influenza. In both cases, a decline in seasonal factors is observed at the end of the year—a pattern not observed in mean temperatures (not shown in the figure; see text for details). The seasonal factors in this figure differ slightly from those in Figure 2 because only data up to 2022 were used for the comparison, whereas Figure 2 presents seasonal factors calculated for the entire study period up to 2023.

Time series decomposition was also conducted for the number of influenza diagnoses in the Masovian voivodeship. It is important to note that only data up to the end of 2022 were used due to incomplete influenza data, as mentioned in the materials section. The trend-cycle component showed that the peak in diabetes diagnoses in 2021 coincided with a decrease in influenza cases. (Figure 3) The seasonal component for influenza diagnoses displayed a similar pattern to that of T1D diagnoses. The peak was observed in winter, the lowest value in summer and a deceleration in growth at the end of the year. The highest seasonal factor, as with diabetes diagnoses, was observed in February (50,256.5), though the lowest was recorded in August (−44,896.8) rather than in June. (Figure 4) Pearson correlation analysis was conducted to compare the seasonality of diabetes and influenza diagnoses. The analysis revealed a very strong correlation between the seasonality of diabetes and influenza diagnoses (*r* = 0.79, *p* = 0.002).

Then, time series decomposition was performed for the average monthly temperatures. The inverse of the temperature data was used for ease of analysis. The trend-cycle component was not analyzed, as temperature data were used to describe environmental factors influencing monthly variations rather than to analyze long-term trends. Therefore, the focus was placed on the seasonal component. The peak was observed in January (seasonal factor 10.88), while June, July, and August had the lowest seasonal factors, with August showing the lowest of the three (−10.197). From autumn through winter, the seasonal factors increased steadily. Unlike the seasonal component observed for both diabetes and influenza diagnoses, there was no pause in its increase at the end of the year. Pearson correlation analysis between the seasonal factors for negative monthly temperatures and diabetes diagnoses showed a strong correlation (*r* = 0.71, *p* = 0.01). We noted T1D onset more frequently in autumn–winter than in spring-summer: 1294 (54%) vs. 1087 (46%) cases (*p*  < 0.0001).

**Figure 5:**
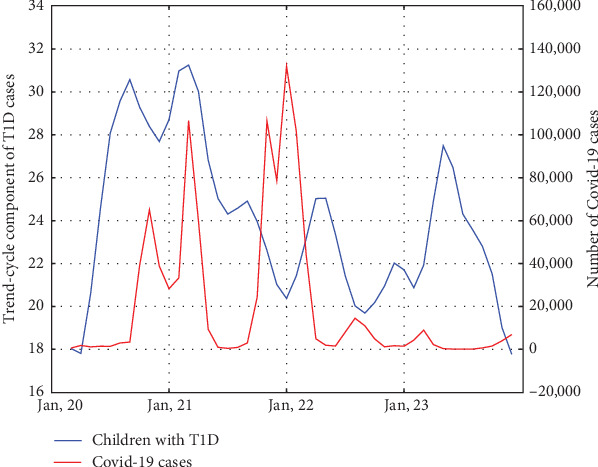
T1D cases and COVID-19 cases during the pandemic period. Two waves with the highest number of COVID-19 cases were identified during the study period. The first occurred between October 2020 and May 2021 and was accompanied by a significant increase in new T1D cases. The second wave, between October 2021 and March 2022, was not associated with an increase in T1D incidence.

Due to the fact that the study period includes the COVID-19 pandemic, we included data on COVID-19 cases in our study. However, we did not perform time series decomposition for these data due to the limitations of the method (which requires at least five complete periods for analysis). We identified two periods with the highest number of SARS-CoV-2 infections in our population. The first wave occurred from October 2020 to May 2021, and the second wave from October 2021 to March 2022. We conducted a Spearman's correlation analysis between the number of infections and the number of T1D diagnoses in both waves. The analysis revealed a strong correlation in the first wave (*r* = 0.96, *p*  < 0.001) and no correlation in the second wave (*r* = 0.086, *p* = 0.87). (Figure 5)

**Figure 6:**
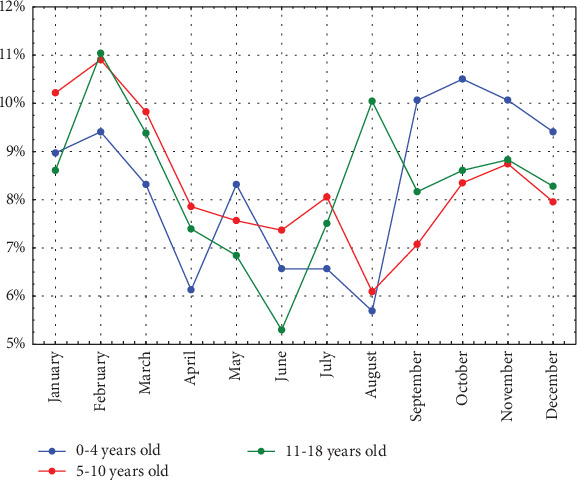
Monthly distribution of T1D diagnoses in different age groups. The figure illustrates the monthly distribution of T1D diagnoses in different age groups expressed as the percentage of cases diagnosed in a given month relative to the total number of patients in each age group. The youngest children (blue line) exhibit a distinct seasonal pattern, with the highest proportion of diagnoses occurring in October (10.5%). In contrast, the peak incidence in the older age groups is observed in February (~11%).

To analyze the differences in seasonality of diagnoses between age groups, we compared the percentages of diagnoses in each month for the 0–4, 5–10 and 11–18 years age groups. The seasonality of diabetes diagnoses appears to differ between these groups. In the youngest children, the peak in diagnoses is observed in October (10.5% of all in this age group). In contrast, the older children's group, due to its large size, seems to have the greatest influence on the overall results for the entire study population. Similar to the time series decomposition analysis for the entire group, the peak in diagnoses in this age group is observed in February (10.9% and 11.04% in the groups of children aged 5–10 and 11–18 years, respectively). (Figure 6)

## 5. Discussion

We observed an upward trend in T1D cases. Between 2015 and 2023, the number of new diagnoses among children in the Masovian Voivodeship increased by approximately 30%. However, after accounting for demographic factors, the increase was less pronounced. Other studies conducted in recent years also report an upward trend [26, 27].

Moreover, seasonality in T1D incidence was noted, with a peak in February and the lowest number of new cases in June. Similar to our previous results [6], the present study showed a seasonal pattern in the incidence of T1D with fewer children diagnosed in spring–summer and a higher incidence in autumn–winter. This finding is consistent with the results of other studies on the seasonality of T1D incidence [1–6, 9, 10]. There are differences between studies regarding the specific months reported as having the highest and lowest incidence of T1D. For example, one Polish study reported a peak in March [27], our previous study in January [6]. However, the general pattern (i.e., higher incidence of T1D during the colder months) appears to be a well-established phenomenon, and our study further supports this finding.

Our analysis revealed that the seasonal variation of T1D incidence does not follow a purely sinusoidal pattern. In addition to the peak incidence in February and the lowest number of new cases in June, we observed that T1D incidence does not increase linearly between June and February. Instead, there is a noticeable decline in new cases in December. A deceleration in the increasing incidence of T1D in December has been previously reported [28]. In that study, the authors suggested that the lower number of T1D diagnoses in December, compared to November and January, resulted from the holiday period. This remains a reasonable hypothesis, since The Health Needs Map [29] of the Masovian Voivodeship indicates a significant decrease in general pediatric and specialist hospitalizations in the second half of December. However, it remains unclear whether this is due to limited access to healthcare facilities during this period or reflects a lower incidence of respiratory tract infections. The latter is also possible, as influenza cases exhibit a similar seasonal pattern, as described below.

It is speculated that seasonality in diabetes incidence may be caused by viral infection. Since our study focuses on viral infections as potential contributors, we compared the seasonal patterns of T1D incidence with historical monthly temperature data and historical influenza incidence data. It is important to clarify that our objective was not to demonstrate a direct link between influenza and the observed seasonality of T1D. However, we considered influenza incidence data as an indicator for seasonal population-wide exposure to other respiratory viruses, given that the transmission patterns of influenza virus are similar to those of other respiratory tract infections.

Some studies propose a potential role of influenza virus infection in the development of T1D [30–32], but this effect appears to be weak and has been observed primarily in cases of pandemic influenza A (H1N1) [31, 33, 34]. To further explore this hypothesis, in addition to comparing the seasonal factors of influenza incidence and T1D cases, we also analyzed the trend-cycle components of these two datasets. Our analysis revealed a very strong correlation between the seasonality of diabetes and influenza diagnoses. Additionally, it appears to better reflect the seasonality of T1D incidence than average monthly temperatures. This suggests that factors beyond those directly linked to seasonal factors may contribute to the observed pattern of T1D incidence. However, the fact that the peak incidence of T1D coincides with the period of lowest influenza incidence suggests that T1D seasonality is associated with seasonal viral infections but does not result directly from influenza infection. This observation is supported by a study in which no impact of influenza infection on the appearance of islet autoantibodies was found [35]. However, it is possible that the infection accelerates the onset of clinical symptoms. This has been particularly suggested in cases of more severe infections [34]. The decline in influenza cases in 2020 was likely caused by the lockdown due to the SARS-CoV-2 epidemic. During this period, schools were closed, and children's contact with their peers was reduced, limiting the spread of common viral infections. Nevertheless, during this time, we observed the highest peak in T1D cases, clearly indicating the role of SARS-CoV-2. The possible association between COVID-19 and the development of T1D is discussed in detail in the following sections.

The comparison between the expected and observed number of new T1D diagnoses revealed a lower than expected incidence in March 2020, a higher than expected incidence in February 2021, and a lower than expected incidence in October 2023. We associate the low number of newly diagnosed cases in March 2020 with the onset of the COVID-19 pandemic and the implementation of lockdown measures. A similar observation was reported in the analysis of the SWEET database [22]. We associate the higher than expected number of diagnoses in February 2021 with the increased incidence of COVID-19, as described below. We did not identify a potential cause for the lower than expected number of cases in October 2023, but it was also reported in another study [36].

We identified two local waves of COVID-19 infections (the first wave between October 2020 and May 2021, and the second wave between October 2021 and March 2022). The peak of new T1D cases occurred during the first wave but was not observed during the second wave. The impact of SARS-CoV-2 infection on the development of T1D has been extensively studied, and recent meta-analyses on this topic have been published [37–39]. They conclude that COVID-19 infection is associated with an increased risk of developing T1D. A recent study suggests that T1D incidence has returned to pre-pandemic levels [40], which is consistent with our observations. It has been demonstrated that SARS-CoV-2 infection can accelerate the onset of diabetes symptoms in individuals with preexisting islet autoantibodies [41]. It is also known that SARS-CoV-2 infection increases the risk of autoimmunity in genetically predisposed patients [42]. However, it remains unclear why the first wave of the COVID-19 pandemic was more strongly associated with the development of T1D than the second wave. Probable explanations for this observation are described in the following sections.

One possible explanation could be related to different dominant viral variants during these two waves. At the turn of 2020 and 2021 (first wave in our dataset), the Alpha variant of SARS-CoV-2 was dominant in Poland. Then, throughout the second half of 2021 (second wave in our dataset), the Delta variant was dominant. Since 2022, the Omicron variant has been the predominant cause of COVID-19 in Poland [43]. Autoimmune diseases were observed more frequently during the Delta and pre-Delta periods than during the period dominated by the Omicron variant [44]. We observed an increase in T1D incidence during the period when the Alpha variant of SARS-CoV-2 was dominant.

Another possible explanation is that as the pandemic progressed, population immunity increased due to prior vaccination or infection.

In Poland, free COVID-19 vaccinations were introduced in May 2021 for adolescents aged 16–17, in June 2021 for children aged 12 and older, in December 2021 for children aged 5–11 and in December 2022 for children aged 6 months to 4 years. In 2022, in the Masovian Voivodeship, 12.9% of children aged 0%–9% and 25.4% of children aged 10–17 were vaccinated [45]. These data indicate that during the first wave of COVID-19, no children were vaccinated. During the second wave vaccination coverage remained low, suggesting that COVID-19 vaccination had minimal impact on the incidence of T1D. Consistent with our observations, Mameli et al. [46] recently reported no association between COVID-19 vaccination in children and the onset of T1D. However, the impact of the COVID-19 vaccine on the development of T1D remains unclear. Currently, the AntiViral Action against T1D Autoimmunity (AVAnT1A) study is being conducted to evaluate the effect of COVID-19 vaccination in children from 6 months of age on the incidence of T1D [47].

Low COVID-19 vaccination coverage during the study period indicates that infection played a major role in shaping immunity in the pediatric population. It is well established that previous SARS-CoV-2 infection reduces the risk of severe disease during reinfection [48]. This would suggest that the effect of viral infections on the onset of T1D symptoms depends not only on the specific virus involved but, more importantly, on the strength of the immune system's response. A similar relationship has been proposed for other viral infections [17], and it is possible that a comparable mechanism applies to COVID-19 as well.

Given the growing evidence for the role of viral infections in triggering or accelerating autoimmunity, improving vaccination coverage may be an important public health strategy to prevent severe courses of infections potentially involved in the development of T1D. Beyond COVID-19, vaccination coverage rate for recommended vaccines among children in Poland remains very low. For example, influenza vaccination coverage among children up to 14 years of age, did not exceed 5% between 2013 and 2023 [49]. According to the International Society for Pediatric and Adolescent Diabetes (ISPAD) [50], children with diabetes should follow the same recommended vaccination schedule as their healthy peers.

Regarding differences in the seasonality of T1D incidence across age groups, the youngest children (aged ≤4 years) exhibited a distinct seasonal pattern, with a peak in October. Some studies have also reported a different seasonality pattern in this youngest age group [5, 6, 9]. One potential factor involved in T1D pathogenesis in this group is intrauterine infections [13], although this does not fully explain the observed seasonality in this age group. Another possible explanation is a faster progression of autoimmunity following enteroviral infection in young children, as enteroviral infections also peak in the summer and early autumn in temperate climates [51]. The frequency of enterovirus infections increases in the autumn with the start of preschool attendance.

The limitation of our study is the use of influenza incidence as a proxy for the circulation of seasonal respiratory viruses. While influenza trends can reflect general patterns of respiratory virus activity, other seasonal pathogens, such as respiratory syncytial virus (RSV), adenovirus, or parainfluenza may also play a role in triggering T1D onset. This assumption may not fully account for the complexity of viral exposures and should be considered when interpreting the findings.

## 6. Conclusions

Our confirmation of a predictable seasonal pattern in T1D incidence, with a peak in February, underscores the value of health forecasting for planning diabetes care. The analysis of viral infections during the study period demonstrated an association between SARS-CoV-2 infection and the development of T1D, but only during the first wave. The seasonality of T1D incidence is closely correlated with the seasonal pattern of influenza cases. However, the overall trends in influenza and T1D incidence differ. The observed seasonality in diabetes diagnoses is most likely driven also by seasonal viral infections transmitted via mechanisms similar to those of the influenza virus. Further research is recommended on the impact of influenza and SARS-CoV-2 vaccinations on the occurrence of T1D in children, as well as the influence of infection severity on the development of symptomatic T1D.

## Data Availability

The data that support the findings of this study are available upon request from the corresponding author. The data are not publicly available due to privacy restrictions.
